# Early diagnosis of acute lymphoblastic leukemia utilizing clinical, radiographic, and dental age indicators

**DOI:** 10.1038/s41598-025-95014-w

**Published:** 2025-04-11

**Authors:** Rehab F Ghouraba, Shaimaa S. EL-Desouky, Mohamed R. El-Shanshory, Ibrahim A. Kabbash, Nancy M. Metwally

**Affiliations:** 1 Oral Medicine, periodontology, Oral Diagnosis, and Radiology Department, Faculty of Dentistry, Tanta University, Tanta, Egypt; 2 Pediatric Dentistry, Oral Health, and Preventive Dentistry Department, Faculty of Dentistry, Tanta University, Tanta, Egypt; 3 Pediatrics Department, Faculty of Medicine, Tanta University, Tanta, Egypt; 4 Public Health & Community Medicine Department, Faculty of Medicine, Tanta University, Tanta, Egypt

**Keywords:** Leukemic child patient, Cone beam computed tomography (CBCT), Age Estimation, Bone density, Cancer, Medical research, Oncology

## Abstract

Leukemic patients often display clinical signs like anemia, thrombocytopenia, and hepatosplenomegaly. Early diagnosis is crucial for intervention and improved prognosis. Dentists can help identify these signs through oral masses, gingival bleeding, and oral ulceration, with radiographical features like bone osteolysis, moth-eating appearance, and abnormal tooth chronology. This study aimed to achieve early diagnosis of leukemic child patients (LCP) by the dentist based on their clinical, age estimation, and radiographical oral signs. Twenty-three children suffer from leukemia, selected after an initial diagnosis based on their clinical signs with an abnormal CBC or abnormal WBCs. These patients were accessed clinically for oral signs and radiographically using panoramic radiographs and cone beam computed tomography (CBCT) to evaluate chronology and bone density. LCP were compared with systematically free control child cases (SFC) who went for a panoramic image and CBCT scan needed for their orthodontic problems. Clinical results for LCP revealed (100%) of cases showed gingival bleeding, (87%) of cases showed gingival masses, (83%) of cases revealed aphthous-like ulceration, and (100%) of cases had different grades of mobility related to the lower first permanent molar used as markers for tooth affection. Radiographical results revealed a statistically significant decrease (P value ≤ 0.05) in LCP age revealed by panoramic and CBCT images in comparison with their actual age. Also, there was a statistically significant decrease in bone density shown by LCP regarding selected regions. LCP could be early diagnosed by the dentist through clinical and radiographical indicators. Diagnosing acute lymphoblastic leukemia (ALL) in its early stages remains a significant challenge due to the nonspecific and often subtle nature of initial symptoms. Dental practitioners can bridge the gap between routine dental care and early systemic disease detection, potentially expediting medical intervention and improving outcomes for children with acute lymphoblastic leukemia.

## Introduction

Leukemia is the most frequent malignant tumor, accounting for 30% of all childhood cancers. It is sporadic and has three primary subtypes, including acute lymphoblastic leukemia (ALL)^1^, acute myelogenous leukemia (AML), and chronic myelogenous leukemia (CML). The most prevalent subtype, ALL, accounts for about 80% of cases^2^. Blood progenitor cells with genetic alterations develop into acute leukemia. Alterations in the body lead to unregulated self-renewal potential and developmental arrest of progenitor cells, causing immature cells called blasts to invade the body and extra-medullary locations^3^.

Leukemia symptoms are frequently ambiguous and non-specific^4^. Early detection of leukemia is crucial due to the clonal growth of leukemic blasts in bone marrow, which disrupts normal red blood cell, platelet, and neutrophil formation^2^. Mitchell et al.,^1^ highlighted potential acute leukemia presentations in children, including anemia, thrombocytopenia, and neutropenia. Although leukemic patients typically do not initially experience bleeding, thrombocytopenia can be severe. The oral manifestations of leukemia include petechia, mucosal pallor, oral ulcers, bleeding, and enlarged, inflamed gingiva^5,6^. Additionally, chin numbness, tooth discomfort, aberrant tooth movement without obvious caries, cracked lips, and hemorrhagic bullae on the anterior dorsum of the tongue, buccal and labial mucosa have been seen in some cases^7^.

Many radiographic approaches are available to assess the bone level and density as panoramic and periapical radiographs however, they are considered imprecise with limited vision in two planes which necessitates a more accurate 3D imaging modality represented in cone beam computed tomography (CBCT)^8^.

Estimating age helps the treatment planning, particularly in pedodontics and orthodontics. Somatic development, including skeletal maturity, height, and teeth, has been used to determine age in children and adolescents. Teeth are the most durable portion and experience the least degree of structural change^9^. Dental development in children and adolescents has been approached using several different methods. Demirjian et al.,^10^ created a seven-tooth aging scheme based on eight stages of calcification. Willems et al.,^11^ modified the Demirjian approach and developed new tables from which a maturity score is directly expressed in years. It became more simpler by eliminating the difficult process of converting the maturity score to dental age.

Tooth maturation is commonly assessed using panoramic X-rays, which provide a simultaneous view of all teeth. However, panoramic imaging is a two-dimensional (2-D) modality that only captures the anterior-posterior and superior-inferior dimensions, lacking the axial or bucco-lingual plane. This limitation results in superimposed structures and potential magnification errors due to the distance between the image receptor and the object^12^ with lacking advanced software capabilities to correct such errors^13^. Moreover, despite optimal technique, panoramic images may include duplicated airway shadows in the posterior mandibular region, real shadows, and ghost artifacts from the mandible in posterior areas^14,15^.

Cone-beam computed tomography (CBCT), on the other hand, is a three-dimensional (3-D) imaging technique recognized for its accuracy in diagnostic radiography and its relatively low radiation dose, as it allows for the selection of small regions of interest. The simplicity of the technique also makes CBCT more comfortable for pediatric patients^16^. Compared to panoramic imaging, CBCT offers significant advantages in dental age estimation due to its three-dimensional multiplanar navigation, which facilitates detailed observation of morphological features^17^. By using the smallest field of view (FOV), CBCT achieves higher resolution and lower radiation exposure while providing a comprehensive dataset for interpretation^18^.

Additionally, CBCT benefits from advanced software that enables interactive visualization of data, allowing simultaneous evaluation of axial, sagittal, and coronal sections alongside the entire scanned volume^19^. The European Commission Guidelines for CBCT in dental and maxillofacial radiology advise against its routine use in daily dental practice unless there is appropriate justification and optimal image optimization^20^. Oenning et al.^21^ introduced the ALADAIP principle (‘As Low as Diagnostically Acceptable being Indication-oriented and Patient-specific’), emphasizing the importance of patient-centered imaging practices. This approach establishes a new standard in dental imaging, prioritizing clinical needs and individualized patient care^22^.

The purpose of this research was to achieve early diagnosis of leukemic child patients (LCP) by the dentist by reviewing the initial clinical oral manifestation, age estimation, and oral image-related signs of leukemia in children.

## Materials and methods

### Study setting and ethical consideration

This study was conducted as an observational cross-sectional study. It was carried out at the hematology unit, Pediatric Clinic, Faculty of Medicine, also, the dental examination was conducted at the outpatient clinic of the Pediatric Dentistry Department, Faculty of Dentistry, Tanta University. The panoramic and cone beam x-ray was done at the Oral Medicine, Periodontology, Oral Diagnosis & Oral Radiology Department, Faculty of Dentistry, Tanta University. The trial was registered at ClinicalTrials.gov identifier NCT04756258. Approval for this research was obtained from the Faculty of Dentistry, Tanta University Research Ethics Committee, code (#R-OMPDR-1-20-2) following the ethical guidelines outlined in the 1964 Helsinki Declaration and its subsequent revisions. The purpose of the present study was explained to the patient’s parents, who signed an informed consent form.

### Sample size calculation

The sample size and power analysis were calculated using the Epi-Info software statistical package created by the World Health Organization and Centre for Disease Control and Prevention, Atlanta, Georgia, USA version 2002 (https://www.cdc.gov/epiinfo/index.html). The criteria used for sample size calculation with a 95% confidence limit and 80% power of the study. The estimated sample size(n) was 23 patients for each group.

### Eligibility criteria

A total of 50 acute lymphoblastic leukemic children with an age range of 6–10 years who were referred to the outpatient clinic of the Pediatric Dentistry Department, Faculty of Dentistry, Tanta University, requiring diagnosis and management of any dental problems before starting their oncology treatment, were enrolled and evaluated using the study’s inclusion & exclusion criteria. The included leukemic children were selected after an initial diagnosis with ALL confirmed by complete blood picture, bone marrow examination, and immunophenotyping assessed by flow cytometry from the Haematology Unit of the Faculty of Medicine at Tanta University. Acute lymphoblastic leukemic children who started treatment were excluded from the study. Accordingly, twenty-seven patients were excluded and the end-study sample included 23 children. Although the hematology unit achieves a definitive diagnosis through a blood picture and bone biopsy, these children were examined by a panoramic radiograph and CBCT scan to reveal signs of bone affection to guide dentists in the future for early diagnosis of such cases.

**Fig. 1:**
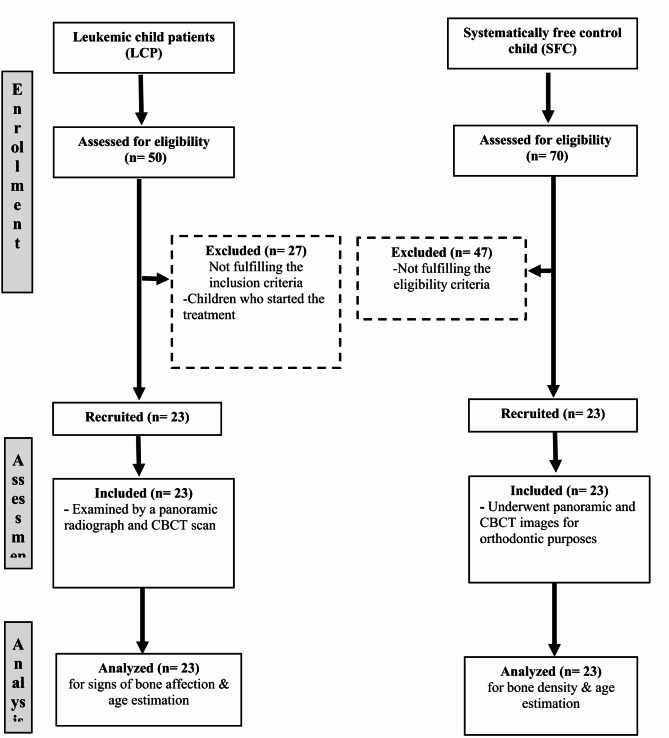
Consort flow chart of the study.

In addition, twenty-three normal child patients out of seventy children with the same age range were chosen as a control group from the Orthodontic Department at Tanta University’s Faculty of Dentistry. These children underwent evaluation for orthodontic proposals using panoramic and CBCT images. Figure 1 shows a consort flow chart with enrollment, assessment, and sample analysis.

### Clinical evaluation

Acute lymphoblastic leukemic children were examined for oral signs such as gingival bleeding, gingival masses, aphthous-like ulceration, and teeth mobility. Tooth mobility of the permanent lower first molars was evaluated using the following score: M0: physiological mobility, M1: slightly increased mobility, M2: definitive, meaning there was no impairment of function, and M3: extreme mobility, meaning the tooth was loose and would not be compatible with function^23^.

### Radiographic evaluation

**Fig. 2:**
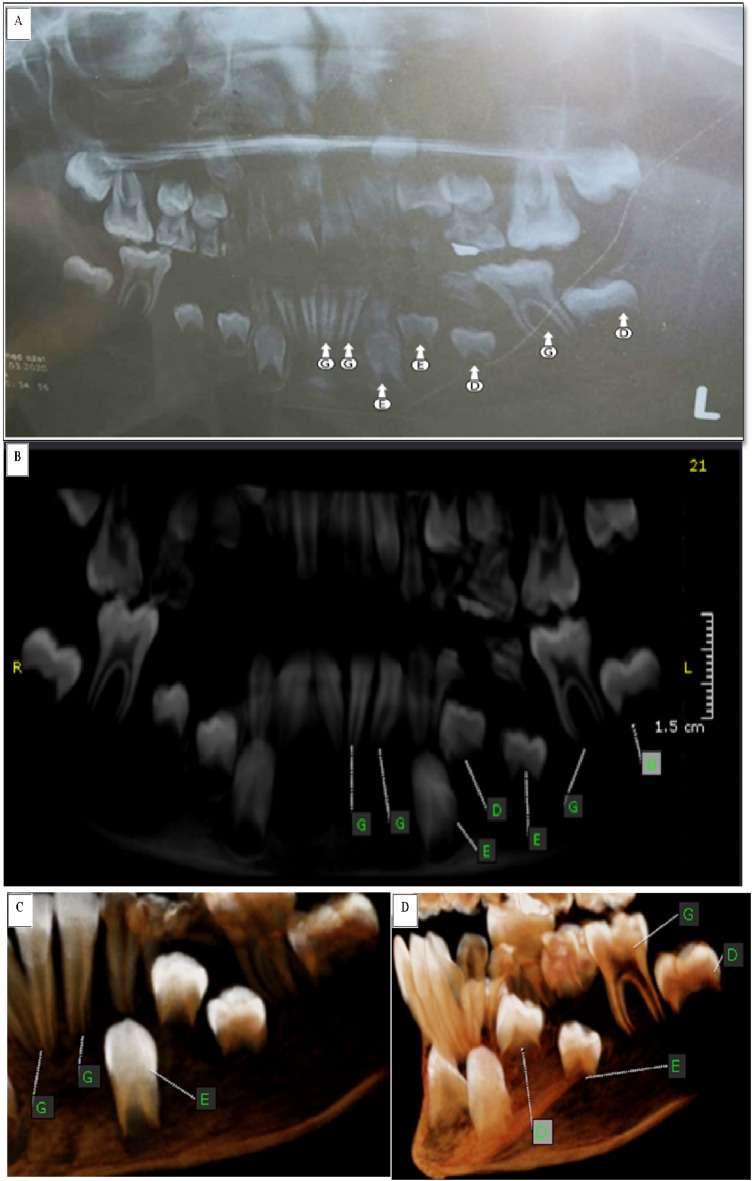
(**A**) panoramic radiograph of a 9-year-old acute lymphoblastic leukemic male child showing different stages of developing lower left seven permanent teeth except for the third molar, scored according to Willems method. (**B**) a reconstructed panoramic view and (**C**, **D**) 3-D (teeth view) of CBCT showing some changes in scoring for the same teeth.

All selected children were examined using fixed exposure parameters by panoramic radiographs (Dental X-Ray Sirona Gmbh-Germany). Furthermore, these children were scanned by CBCT (KaVo OP 3DVision, Kavo Dental, Biberach, Germany) quick scan+(ultra-low dose) with fixed exposure parameters (90 Kv, 3 mA, and0.6 mm voxel size) and the same field of view (16D × 13 H) to achieve standardization and minimal radiation exposure following the concept as low as reasonably achievable (ALARA) supported by American Dental Association^24^. The CBCT images were obtained by using On-Demand Dental software (version 1.0.10.7462), × 64 Edition, copyright 2004–2017 Cybermed, Korea, and license key 670,094,709) (https://ondemand3d.com/en/contents.html?pageId=G1T729Y93EKDQY81877T) as shown in Figure 2.

**Fig. 3:**
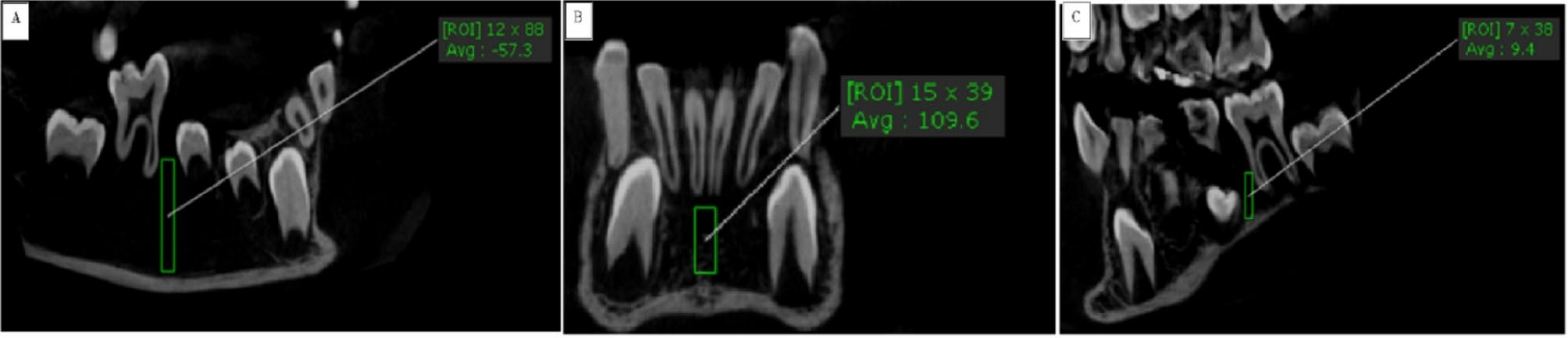
(**A**) sagittal view represents the bone density mesial to the right first permanent molar. (**B**) a coronal view represents bone density between the lower two central incisors. (**C**) sagittal view represents bone density mesial to the lower left first permanent molar.

The bone density was calculated using the ROI of OnDemand software to measure density at the midline region below the lower central incisors while obtaining a coronal view midway between the buccal and lingual cortex achieving standardization and avoiding the lingual cortex that contained genial tubercle that may increase the bone density leading to false positive results in LCP. Also, density in the posterior region of the lower jaw was measured from the mesial region to the lower right and left first permanent molars, then these values were totaled and divided by two. The bone density was measured at the same selected points in leukemic and healthy children to enable accurate comparisons. In addition, the subperiosteal bone reaction was examined in both groups (Fig. 3).

### Dental age assessment

For both groups, the participant’s sex was identified, also the chronological age was determined by deducting their birthdate from the radiographic date, and the researcher was blinded to their actual age. Panoramic radiographs were assessed using computer-aided drafting software (Adobe Photoshop CC 2020, Adobe Systems integrated, San Jose, CA, USA) (https://windowstan.com/software/photoshop-cc-2020/ ), and the CBCT images were evaluated using a 3D module of On Demand Dental software (version 1.0.10.7462), × 64 Edition, copyright 2004–2017 Cybermed, Korea, and license key 670,094,709) (https://ondemand3d.com/en/contents.html?pageId=G1T729Y93EKDQY81877T) to estimate the participant’s dental age using Willems’ method^11^. In each panoramic and CBCT image (Figs. 2 and 3), the seven mandibular left permanent teeth, except the third molar, were assessed for the degree of tooth calcification and scored ‘0’ for no calcification and ‘A’ to ‘H’ for varying degrees of calcification according to the written score descriptions of Demirjian et al.,^10,25^ method as presented in Table 1 since Willems’ method depends in dental age estimation on the tooth’s developmental scores of Demirjian method.

All scores of each mandibular left permanent tooth from the central incisor to the second molar except the third molar were recorded; each score corresponds to a dental age fraction (in years) according to the tables of Willems’ method^11^ for boys and girls. Summing the scores of the seven left mandibular teeth directly resulted in the estimated dental age.

Comparing the estimated Willems dental age with the participant’s chronological age indicated delayed or accelerated dental maturity.

**Table 1:** An explanation of the Tooth’s developmental scores^25^.

Stage	Description
A	A beginning of calcification is seen at the superior level of the crypt in the form of cones. There is no fusion of these calcified points
B	Fusion of the calcified points forms one or several cusps, giving a regularly outlined occlusal surface
C	Enamel and dentin formation is complete at the occlusal surface and converges at the cervical region Dentin deposition is seen. The outline of the pulp chamber has a curved shape at the occlusal border
D	The crown formation is completed down to the cementoenamel junction. The superior border of the pulp chamber in uniradicular teeth has a definite curved form; the projection of pulp horns gives an umbrella top. In molars, the pulp chamber has a trapezoidal form. The beginning of root formation is seen in the form of a spicule
E	*Uniradicular teeth* The walls of the pulp chamber form straight lines, whose continuity is broken by the pulp horn The root length is less than the crown height. *In molars* Initiation of radicular bifurcation is seen as a calcified point or a semi-lunar shape Root length is less than crown height
F	*Uniradicular teeth* The walls of the pulp chamber form an isosceles triangle. The apex ends in a funnel shape. The root length is equal to or greater than the crown height. *In molars* The bifurcation has developed down to give the roots a distinct outline with funnel-shaped endings Root length is equal to or greater than crown height
G	The walls of the root canal are now parallel and its apical end is partially open (distal root in molars)
H	The apical end of the root canal is completely closed. Periodontal membrane has a uniform width around the root and apex

### Statistical analysis

All data was collected, tabulated, and statistically analyzed using Statistical Package for Social Studies (SPSS) version 19 produced by IBM, Illinois, Chicago. Numerical variables were presented as a range, mean, and standard deviation and a comparison of mean values between leukemic and control groups was tested using an unpaired student’s t-test. The comparison of tooth mobility between groups was conducted using the Monte Carlo exact test. Comparison of mean values of the chronological age, CBCT dental, and panoramic dental ages were done using one-way analysis of variance (ANOVA). When the results of ANOVA were found statistically significant, pairwise analysis was performed using the Bonferroni test. For categorical variables, the number and percentages were calculated and the differences between observations of subcategories were performed using Fisher’s exact test. The significance level was set as a P value ≤ 0.05.

## Results

Table 2 shows the gender distributions of the enrolled children in each group. 43.5% of the LCP group were male, 56.5% were female, and in the healthy children group (SFC), 47.8% were male, and 52.2% were female.

**Table 2:** Demographic distribution of study participants.

	Total *N*	LCP *N*	Control (SFC) *N*
Sex			
Male	21 (45.6)	10 (43.5)	11 (47.8)
Female	25 (54.4)	13 (56.5)	12 (52.2)

### Clinical picture

All acute lymphoblastic leukemic cases (100%) showed gingival bleeding, 20 LCP cases (87%) showed gingival masses, and 19 LCP cases (83%) revealed aphthous-like ulcerations. In addition, 15 LCP cases (65%) showed grade III mobility related to permanent lower first molars while eight cases (35%) showed grade II mobility that was used as markers for tooth affection (Table 3).

**Table 3:** Comparison of mobility scoring related to permanent lower first molars among leukemic child patient (LCP) and control groups.

Mobility score	Leukemic group (LCP) (*n* = 23)		Control group (SFC) (*n* = 23)	
	*n*	%	*n*	%
M0	0	0.0	23	100
M1	0	0.0	0	0.0
M2	8	35.0	0	0.0
M3	15	65.0	0	0.0

*Significant Mone Carlo exact test (*p* < 0.001).

### Bone density evaluation

The mean values of bone density at midline and mesial to lower first permanent molars were (256.91 *±* 71.98 and − 66.5 *±* 33.5), and (572.06 *±* 44.15 and 444.13 *±* 17.53) for the leukemic child patients (LCP) group and control group, respectively. Upon using the unpaired t-test, there was a statistically significant decrease (*P* ≤ 0.05) in bone density at midline, and mesial to lower first permanent molars in the LCP group in comparison to the control group, suggesting severe osteoporosis in LCP, as shown in Table 4; Figure 3.

The subperiosteal bone reaction was found in twenty cases (87%) in LCP compared to two cases (9%) in the control group. Using the Fisher exact test, there was a statistically significant difference (*P* ≤ 0.05) suggesting a positive subperiosteal bone reaction in LCP, as displayed in Table 5.

### Dental age estimation

Concerning age estimation using the Willems’ method in leukemic child patients (LCP) revealed that the mean values for chronological age, and dental age estimated using panoramic and CBCT images were 8.10 ± 1.25, 7.27 ± 1.29 and 6.771 ± 1.339 respectively. Using repeated one-way ANOVA, there was a statistically significant difference (*P* ≤ 0.05). Upon using Bonferroni’s test, there was a statistically significant decrease (*P* ≤ 0.05) in dental age estimated by panoramic and CBCT in comparison with chronological age, suggesting under-estimation of the dental age in the LCP group as shown in Table 6.

Regarding age estimation using the Willems’ method in the control group showed that the mean values for chronological age and dental age estimated on panoramic and CBCT images were 8.01 ± 1.09, 7.68 ± 1.04, and 8.02 ± 0.97 respectively. Using repeated one-way ANOVA, there was a statistically significant difference (*P* ≤ 0.05). When using Bonferroni’s test, there was a statistically significant decrease (*P* ≤ 0.05) between the dental age estimated on panoramic when compared with chronological age. Furthermore, there was no statistically significant difference (*P* > 0.05) between the dental age estimated using CBCT and chronological age, as presented in Table 6.

The inter-group comparison revealed that using the unpaired t-test, there was no statistically significant difference (*P* > 0.05) regarding chronological age and dental age estimated using panoramic radiographs between both groups while there was a statistically significant difference (*P* ≤ 0.05) regarding dental age estimated using CBCT images between both groups, as illustrated in Table 6.

**Table 4:** Inter-group comparison of mean bone density at midline and mesial to lower first permanent molars among leukemic child patient (LCP) and control groups.

Bone density	Leukemic group (LCP)	Control group (SFC)	t	*p*₪
At midline			17.898	< 0.001***
Range	112–377	488.5-675.3		
Mean ± SD	256.91 + 71.98	572.06 + 44.15		
Mesial to lower first molar			121.103	< 0.001***
Range	-66.5 + 33.5	399–489		
M ± SD	-53.16 + 8.97	444.13 + 17.53		

(p₪ ) un Paired t test Significance: p*<0.05, p**<0.01, p***<0.001.

**Table 5:** Inter-group comparison of sub-periosteal bone reaction among leukemic child patient (LCP) and control groups.

Sub-periosteal bone reaction	Presence		Absence	
	*N*	%	*N*	%
LCP	20	87%	3	23%
Control (SFC)	2	9%	21	91%
P~	0.0001***			

P ~ Fisher exact test Significance: p*<0.05, p**<0.01, p***<0.001.

**Table 6:** Comparison of mean panoramic and CBCT dental ages using Willems method for leukemic child patient (LCP) and control groups.

Age in years	Leukemic group (LCP)	Control group (SFC)	t	*p*₪
Chronological age			0.256	0.799
Range	6.0–10.0	6.1–10.0		
Mean ± SD	8.10 *±* 1.25	8.01 *±* 1.09		
Panoramic dental age			1.187	0.242
Range	4.9–9.3	6.0-9.3		
Mean ± SD	7.27 *±* 1.29	7.68 *±* 1.04		
CBCT dental age			3.626	0.001*
Range	4.4–8.9	6.3–9.8		
Mean ± SD	6.77 *±* 1.34	8.02 *±* 0.97		
F	253.994	18.029		
P^#^	< 0.001***	< 0.001***		

(p₪) unpaired t-test, (P#) Repeated one-way ANOVA: p*<0.05, p**<0.01, p***<0.001.

Each measure was significantly different from the other two.

Panoramic age was significantly different from chronological and CBCT ages.

## Discussion

Unfortunately, the majority of research that has been published has only looked at the clinical oral signs of oral cancer, occasionally including a panoramic radiography evaluation of adults or children. However, the primary goal of this study was to diagnose leukemia as early as possible using clinical judgment, age estimation, and CBCT radiographic examination. The first diagnosis of leukemia was based on clinical signs and symptoms that support the diagnosis. But there are existing diagnostic challenges concerning many complementary procedures, like flow cytometry, cytogenetic evaluation, and bone marrow biopsy, which are used to arrive at the final diagnosis^26^. The primary drawback of flow cytometry is its inability to correlate with histomorphology. Furthermore, flow cytometry requires fresh, viable samples, and the reduced viability of certain neoplasms often hinders accurate analysis^27^. Also, in routine clinical practice, obtaining cytogenetic test results can be challenging and time-consuming, especially in non-academic centers, where delays may arise due to infrastructure limitations, such as insufficient laboratory facilities and a lack of specialized personnel in pathology and genetics^28^. Bone marrow diagnosis is complex, requiring pathologists to distinguish subtle differences between neoplastic conditions by combining insights from morphological evaluations with results from ancillary tests^29^. An early diagnosis improves the prognosis and increases the patient’s probability of survival by enabling prompt initiation of antineoplastic treatment^30^. Leukemia’s initial warning signs and symptoms may appear in the mouth or neck and a simple dental examination may reveal them^31^. Once they are recognized, the dentist should respond by recommending further testing or directing the patient to other specialists^32^.

The acute lymphoblastic leukemic children in this study suffered from different oral manifestations such as gingival bleeding, gingival mass, aphthous-like ulceration, and different grades of mobility related to permanent lower first molars. These findings align with numerous other research investigations^33–36^. Also, Ponce-Torres et al.,^37^ found that children with acute lymphoblastic leukemia have an increased risk of gingival inflammation when compared to healthy children.

The most prevalent clinical problem affecting hard tissues in this observational study was teeth mobility. CBCT and panoramic radiography were utilized to assess the bone and dental structures. This manifestation was also reported by Quispe et al.^38^, who stated that the radiographic scans mostly showed substantial vertical bone loss, cortical enlargement of the alveolar bone, osteolytic regions, and thickening of the bone surrounding the teeth with mobility. These previously discussed primary consequences are those that arise primarily from the disease itself also, gingival or tooth problems occur by the infiltration of malignant cells into oral structures like the tooth pulp, gum, and bone^39^.

Willems’ method was selected for dental age assessment in the current study because it is based on the Demirjian method and has clear, straightforward criteria based on the form and proportion of root length^40^. Seven teeth from the left quadrant of the jaw were used in the current study to estimate dental ages because they show the age range at which root calculations begin and end, which is similar to the age range of the study participants^41^.

In the majority of studies, age estimation was measured in acute lymphoblastic leukemia and other malignancies receiving chemotherapy, there were detrimental effects of the chemotherapeutic drug on the development of the skeleton and teeth^42–45^. Unlike other studies, this investigation was done before antineoplastic therapy. This study revealed that there was a statistically significant decrease in dental age estimated by panoramic and CBCT in comparison with chronological age suggesting underestimation in the LCP group. The reason behind this could be pulp infiltration with cancerous cells infiltrating oral tissues like the gum and bone as stated by Soares and coworkers^39^, potentially influencing root growth, and leading to delayed root closure. Moreover, Almonaitiene et al.,^46^ pointed out that underlying medical conditions and malignancy may impede dental maturity.

This study showed that there was a statistically significant decrease in dental age estimated on panoramic images compared to that estimated on CBCT in the control group (SFC). Furthermore, there was no statistically significant difference between dental age estimated using CBCT and chronological age, suggesting that CBCT is a more reliable method than a panoramic radiograph. These results agreed with Merdietio Boediand et al.,^17^ who conducted a meta-analysis to look into the relationship between dental (DA) and chronological (CA) ages, as well as the reliability of dental age estimation techniques in cone beam computed tomography (CBCT). In their conclusion, they found strong evidence supporting the reproducibility and reliability of CBCT methods for estimating dental age. Furthermore, Yousefi et al.^47^ indicated that CBCT is a valid and consistent method for estimating dental age.

Furthermore, because preliminary laboratory and clinical data could not be precise, imaging tests in patients with bone pain and extramedullary involvement may provide the clinician with substantial additional information^48^. It is well known that CBCT has better diagnostic accuracy than panoramic radiographs due to avoiding panoramic errors even with ideal technique without patient position errors such as real airway shadows and ghost shadows of posterior jaw regions which may lead to inaccurate density interpretation with panoramic radiographs^49^. However, it should be taken into consideration the inferiority of CBCT in measuring bone density in comparison to computed tomography (CT) also, the higher liability of CBCT for scatter radiation^50,51^ and partial volume artifact^52^ with increasing field of view. However, CBCT was used in this study due to ease of interpretation and the familiarity of the dentist with CBCT. In addition, the CBCT (KaVo OP 3DVision, Kavo Dental, Biberach, Germany) machine allows the chin rest position with software properties of pseudo-cyclic rotation before making a real cycle of exposure eliminating the child’s liability for claustrophobia and fear.

In our study, there was a statistically significant decrease in bone density in the LCP group compared to the control group; it suggests the presence of severe osteoporosis, which is considered a severe loss in bone mineral density leading to loss of bone supportive function that was evident by tooth mobility. This may be attributed to the infiltration of leukemic cells in bone marrow and the inability to perform proper nutrition^53^. These results were in agreement with Athanassiadou and coworkers^54^, who conducted a study to seek a correlation between lumber spine bone density measured by dual-energy X-ray absorptiometry among acute lymphoblastic leukemic children during their period of consolidation. They revealed reduced bone density in acute lymphoblastic leukemic children in comparison with the healthy group, with eight children out of twenty children in the acute lymphoblastic leukemic group having osteopenia. The present study findings revealed that CBCT is considered reliable for bone density measurement in the LCP; this is in line with Leonard et al.,^55^ who stated that bone mineral density measurements are dependent on bone area (length and size) but do not account for bone depth or volumetric density. As revealed by Cowell and Wüster^56^. , , an alternative approach could involve using quantitative computed tomography (CT) to evaluate bone density more comprehensively; however, this method involves significantly higher exposure to ionizing radiation and limited accessibility.

There was a statistically significant subperiosteal bone reaction in LCP in the present study; this may be explained by the fact that the fast-growing tumor has the potential to destroy cancellous bone and quickly approach cortical bone, inducing periosteal reactions to enclose the tumor and restore bone^57^. These results were in agreement with Abbas et al.,^58^ and Hafiz et al.,^59^ who discussed a case report of a child with acute lymphoblastic leukemia and reported that both cases had lamellated subperiosteal reactions with diffuse osteolytic lesions.

To the best of our knowledge, this is the first study using oral manifestation, dental age estimation, and radiological examination as combined indicators for the early identification of acute lymphoblastic leukemia. The present study has some limitations including a restricted age range of children and the selection of acute lymphoblastic leukemia as a subtype of leukemia in children. Also, despite the sample size and power analysis for this study being calculated, there is a need to perform the study on a relatively larger sample size to strengthen the validation of results. Future studies encompassing a wider age spectrum, large sample size, and incorporating other leukemic subtypes could provide more comprehensive insights into the utility of clinical, radiographic, and dental age indicators in early diagnosis. Also, further clinical research with a longer follow-up time that takes into account neoplastic therapy and the recovery period is needed.

## Conclusions

Dental professionals could diagnose leukemic children at an early age due to their clinical symptoms, which included gingival bleeding, severe tooth mobility, high risk of gingival mass, and aphthous ulceration. Additionally, radiographic signs indicated underestimation by panoramic and CBCT images, along with severe osteoporosis measured by CBCT images. Furthermore, when estimating a child’s age, CBCT is considered more accurate and reliable than panoramic estimation.

## Data Availability

On reasonable request, the datasets utilized and/or analyzed during the present study are accessible from the corresponding author.

## Declarations

The Helsinki Declaration of 1964 and its later amendments were complied with, and the ethical committee^46^ of Tanta University’s Faculty of Dentistry granted ethical approval for this study under code (#R-OMPDR-1-20-2). The patients’ guardians were told about the goal of the study and an informed consent form was signed before clinical procedures.
